# Temporal genetic differentiation in *Glossina pallidipes* tsetse fly populations in Kenya

**DOI:** 10.1186/s13071-017-2415-y

**Published:** 2017-10-10

**Authors:** Winnie A. Okeyo, Norah P. Saarman, Michael Mengual, Kirstin Dion, Rosemary Bateta, Paul O. Mireji, Sylvance Okoth, Johnson O. Ouma, Collins Ouma, Joel Ochieng, Grace Murilla, Serap Aksoy, Adalgisa Caccone

**Affiliations:** 1 Yale School of Public Health, Yale University, New Haven, CT USA; 2 Biotechnology Research Institute, Kenya Agricultural and Livestock Research Organization, Nairobi, Kikuyu Kenya; 3 Department of Biomedical Science and Technology, School of Public Health and Community Development, Maseno University, Kisumu, Maseno Kenya; 4 Department of Ecology & Evolutionary Biology, Yale University, New Haven, CT USA; 5 Centre for Geographic Medicine Research Coast, Kenya Medical Research Institute, Kilifi, Kenya; 6 Africa Technical Research Center, Vector Health International, Arusha, Tanzania; 7 Centre for Biotechnology and Bioinformatics, University of Nairobi, Nairobi, Kenya

**Keywords:** Effective population size, *Glossina pallidipe*s, Microsatellites, Population bottleneck, Temporal changes in allelic frequencies, Vector control

## Abstract

**Background:**

*Glossina pallidipes* is a major vector of both Human and Animal African Trypanosomiasis (HAT and AAT) in Kenya. The disease imposes economic burden on endemic regions in Kenya, including south-western Kenya, which has undergone intense but unsuccessful tsetse fly control measures. We genotyped 387 *G. pallidipes* flies at 13 microsatellite markers to evaluate levels of temporal genetic variation in two regions that have been subjected to intensive eradication campaigns from the 1960s to the 1980s. One of the regions, Nguruman Escarpment, has been subject to habitat alteration due to human activities, while the other, Ruma National Park, has not. In addition, Nguruman Escarpment is impacted by the movement of grazing animals into the area from neighboring regions during the drought season. We collected our samples from three geographically close sampling sites for each of the two regions. Samples were collected between the years 2003 and 2015, spanning ~96 tsetse fly generations.

**Results:**

We established that allelic richness averaged 3.49 and 3.63, and temporal N_e_ estimates averaged 594 in Nguruman Escarpment and 1120 in Ruma National Park. This suggests that genetic diversity is similar to what was found in previous studies of *G. pallidipes* in Uganda and Kenya, implying that we could not detect a reduction in genetic diversity following the extensive control efforts during the 1960s to the 1980s. However, we did find differences in temporal patterns of genetic variation between the two regions, indicated by clustering analysis, pairwise F_ST_, and Fisher’s exact tests for changes in allele and genotype frequencies. In Nguruman Escarpment, findings indicated differentiation among samples collected in different years, and evidence of local genetic bottlenecks in two locations previous to 2003, and between 2009 and 2015. In contrast, there was no consistent evidence of differentiation among samples collected in different years, and no evidence of local genetic bottlenecks in Ruma National Park.

**Conclusion:**

Our findings suggest that, despite extensive control measures especially between the 1960s and the 1980s, tsetse flies in these regions persist with levels of genetic diversity similar to that found in populations that did not experience extensive control measures. Our findings also indicate temporal genetic differentiation in Nguruman Escarpment detected at a scale of > 80 generations, and no similar temporal differentiation in Ruma National Park. The different level of temporal differentiation between the two regions indicates that genetic drift is stronger in Nugruman Escarpment, for as-yet unknown reasons, which may include differences in land management. This suggests land management may have an impact on *G. pallidipes* population genetics, and reinforces the importance of long term monitoring of vector populations in estimates of parameters needed to model and plan effective species-specific control measures.

**Electronic supplementary material:**

The online version of this article (10.1186/s13071-017-2415-y) contains supplementary material, which is available to authorized users.

## Background

Tsetse flies (genus *Glossina)* are the major insect vectors of Human African Trypanosomiasis (HAT) and Animal African Trypanosomiasis (AAT), serious animal and human diseases in sub-Saharan Africa [1–3]. HAT causes fatalities of thousands of people every year, while AAT, also known as Nagana, causes significant mortality in livestock with major economic losses impeding agricultural development [1–3]. Direct prevention and treatment of the diseases has not been possible because no vaccines exist against HAT and AAT, and available drugs for treatment are difficult to administer [1, 4] and drug resistance has been reported [5, 6]. In view of the above, vector control therefore constitutes a major cornerstone of HAT and AAT suppression in Africa. Tsetse control methods include habitat interference, trapping, use of insecticide-treated targets, aerial or ground spraying, insecticide-treated cattle, or the release of sterile/transgenic insects [4, 7–10]. Despite implementation of vector control methods, their success has been limited by the required long timescales and large geographical scales necessary to control tsetse populations. Vector control methods also depend on complex coordination to maximize both their efficiency and long-term monitoring efforts [11].

In Kenya, HAT was first reported in 1902, and expanded during the mid-1950s until control efforts between the1960s and 1980s led to eradication of HAT from most of Kenya [11]. HAT is now limited to a small part of western Kenya along the Ugandan border [11], but re-emergence into regions such as southwestern Kenya remains a critical threat [11–13]. Currently, however, it is AAT that causes the largest threat to human wellbeing through the loss of income**,** when livestock become sick or die of the disease [14]. The major vector of AAT in Kenya is *Glossina pallidipes*, which occurs in savannah, shrub lands, and grassy woodland habitats that match specific conditions of humidity, rainfall and temperature [15, 16]. Suitable conditions occur in non-contiguous patches that are concentrated along the southwestern and coastal borders of Kenya [14], and are at peak suitability during wet seasons [16]. The discontinuous and fluctuating nature of *G. pallidipes* habitat [20] has the potential to cause rapid changes in distribution and population density, and makes the direct monitoring of *G. pallidipes* difficult [15]. One tool that has been helpful in understanding underlying mechanisms of tsetse resurgence after control has been population genetics analyses [4, 17, 18]. Population genetics can thus contribute greatly to our understanding of best practices in vector control and monitoring [17, 18].

To date, population genetics studies of *G. pallidipes* in Kenya have shown high levels of genetic differentiation among micro- and macrogeographical scales [14, 19], but no significant changes in allelic frequencies across short time scales [16]. Previous work found five strong genetic breaks at a macro-geographical scale of hundreds of kilometers [14], and variable but minor differentiation at a micro-geographical scale of 10–30 km [19]. Differentiation across temporal scales has been less well studied. Work by Ouma et al. [16] in southwestern Kenya found no significant changes in allelic frequencies among collections temporally separated by 3–12 months (~2–8 generations) and found that there were signals of recent population bottlenecks [16]. These results suggest there have not been large shifts in allele frequencies in *G. pallidipes*, at least not over short time periods. In contrast, work by Ciosi et al. [20] demonstrated shifts in allele frequencies in populations along the Uganda/Kenya border in samples collected in 2000 that they attributed to a bottleneck resulting from control measures in the 1990s. Thus, temporal shifts in allele frequencies appear to be context dependent, and there is a need for further research to distinguish potential drivers of genetic changes in *G. pallidipes*.

In this study, we use 13 microsatellite loci to describe temporal patterns of genetic variation in *G. pallidipes* populations across longer time scales than previously reported. We focus on two regions of southwestern Kenya, Nguruman Escarpment (Nguruman) and Ruma National Park (Ruma), that share similarities in ecology and evolutionary processes, and a history of successful suppression in the 1960s to the 1980s [21–23] with recent resurgence in tsetse fly numbers [14, 16, 19, 24, 25]. However, these regions differ in land management. Nguruman does not restrict human use and allows grazing, agriculture and fire wood collection [26–29], whereas Ruma is a National Park that strictly prohibits all of these human activities in the interest of wildlife conservation [30]. Here, we evaluate and compare temporal patterns of genetic variation in these two regions, making use of previous collections made in 2003–2009 and a recent collection made for this study in 2015. Our main objectives were to test for signals of reduced genetic diversity lasting from the intensive control efforts during the 1960s to the 1980s, and to determine if there have been statistically significant changes in allelic frequencies between temporally spaced samples made over a period of 12 years (~96 generations). Results add to our understanding of the rate of evolutionary changes that influence *G. pallidipes* population dynamics. This information will help in making empirically derived vector control and monitoring recommendations for these regions in southwestern Kenya that have proven difficult to manage and that are at risk of reemergence of HAT from neighboring regions to the northwest.

## Methods

### Study area and sampling strategy

**Fig. 1:**
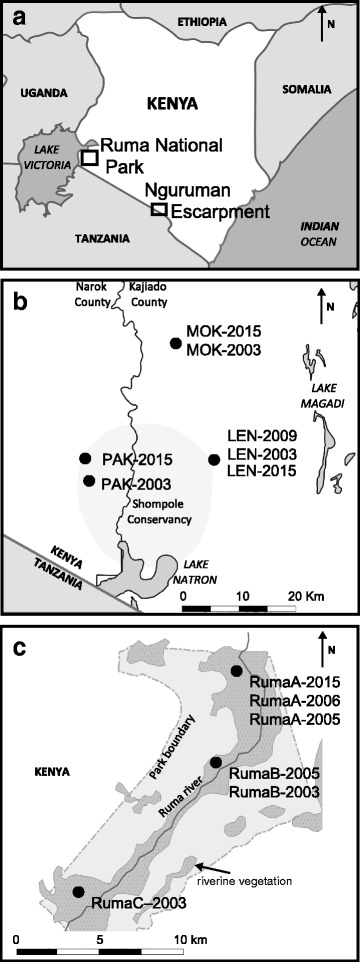
Map of sampled regions southwestern Kenya (**a**), and sampled locations within Nguruman (**b**) and Ruma (**c**). Dots represent the sampling locations and are labeled by their abbreviation in Table 1 and the year of collection

The samples used in this study were collected from Nguruman Escarpment and Ruma National Park in southwestern Kenya (Fig. 1). The Nguruman is a 1500 km^2^ area that lies north of the Kenya/Tanzania border that is managed for community use [31]. Recent control efforts consisted of deployment of 100 NG2B traps, which was reported to have led to a 99% reduction in *G. pallidipes* population density in 1987 [21], but the region has seen recent increase in population density because control programs stopped [14, 16, 19]. The other region of focus in this study, Ruma, is a 120 km^2^ protected area east of the Kenya/Uganda border that is managed for wildlife conservation [30]. Past control efforts included aerial and ground spraying, clearing of underbrush and use of insecticide treated targets from the 1960s to the 1980s [21, 22], but the region has also seen recent increase in population density after control efforts reduced [19, 25].

This study combined samples collected from 2003 to 2009 from past studies [14, 19, 25], and newly collected samples from 2015. In all cases, *G. pallidipes* were collected using bi-conical or *Ngu* traps that were set in clusters of 3–5 traps separated by less than 5 km. Collected flies were preserved in 80% ethanol, transported for a maximum of 5 days at room temperature, and stored at 4 °C. Later, a subsample was chosen for DNA extraction and microsatellite screening. For Nguruman, we screened an average of 38 individuals per site, collected from three locations spaced < 22 km apart (Fig. 1b). For Ruma, we screened an average of 20 individuals per site collected from the same three locations described by Ouma et al. [19] spaced < 14 km apart (Fig. 1c).

### DNA extraction and genotyping

We extracted total DNA from two legs per fly using either the PrepGEM insect DNA (ZYGEM Corp Ltd., Hamilton, New Zealand) or the Qiagen DNAeasy blood and tissue (Qiagen, Hilden, Germany) extraction kits, following the manufacturer instructions. We used the Promega GoTaq kit (Madison, WI, USA) together with fluorescently-labeled primers for a 15 μl PCR reaction as follows: 1 μl of DNA template, 6 μl of distilled H_2_O, 2.6 μl distilled Promega PCR Buffer, 0.1 μl of 100× BSA (molecular grade), 0.5 μl each of 10 mM forward and reverse primers, 1.1 μl of 25 mM MgCl_2_, 1.1 μl of 10 mM dNTP mix, and 0.1 μl of 5 U/μl GoTaq DNA polymerase. Additional file 1: Tables S1 and S2 summarizes the microsatellite genotype strategy and protocol sources [32–35], and the multiplexing conditions. While GpB20b, GpC26b, GpA19a, GpB6b, GpC5b, GpC10b and GpCAG133 were developed for *G. pallidipes* [34] (Additional file 1: Table S1), the other six markers were developed for *G. morsitans* or *G. fuscipes* [32, 33, 35] (Additional file 1: Table S1) and have not all been validated for use in population genetic studies of *G. pallidipes* [32, 33, 35]. Four of these six markers, GmmK22, GmmA06, GmmC17 and GmmL11 [33, 36] have been tested for amplification in *G. pallidipes* [22, 34]. Since the remaining two loci, D05 and Gmm8 [32, 35], had never been used in *G. pallidipes* prior to their inclusion in this study, we tested them in two individuals from four sampling sites using PCR amplification protocols used in other tsetse species [33, 35] to assess their reliability and variability*.* For all markers, PCR products were multiplexed in groups of two or three loci (Additional file 1: Table S2) together with 0.5 μl of the Gel Co ROX internal size and Highly De-ionized Formamide (Gel Company, 665 Third street, San Francisco, CA, USA) to bring the total volume to 15 μl. The PCR products were then sized on an ABI 3730xL automated sequencer at the DNA Analysis Facility on Science Hill at Yale University (http://dna-analysis.yale.edu/). Alleles were scored using the program GeneMarker v 2.4.0 (Soft Genetics, Pennsylvania, USA). For all 13 markers, to ensure repeatability of genotype calls, we manually edited the automatically scored peaks twice independently following agreed upon binning rules (Additional file 1: Table S3), retaining only congruent genotype calls, which made up close to 100% of the peaks with amplification.

### Microsatellite marker validation, genetic diversity, and demographic estimates

For all 13 markers, regardless of their history of use in population genetics studies in *G. pallidipes*, we conducted marker validation analysis to ensure that they did not contain null alleles or violate population genetics analysis assumptions. We tested for presence of null alleles using MICROCHECKER v2.2 [37], and for deviations from Hardy-Weinberg equilibrium (HWE) and for signals of linkage disequilibrium (LD) on a per-locus basis using Genepop v4.1 [38], and corrected for multiple tests with the Benjamini-Hochberg [39] method in both analyses.

To assess patterns of genetic diversity for all samples singly and grouped by time point, we estimated allele frequencies using GenAlex v6.502 [40], and allelic richness using FSTAT v2.9.3.2 [41]. Levels of observed (H_O_) and expected (H_E_) heterozygosity were estimated using Arlequin v3.5 [42]. Inbreeding coefficient (F_IS_) and deviations from HWE expectations were tested using FSTAT v2.9.3 [41] based on 10,000 randomizations. To understand the possible impact of the control efforts during the 1960s to the 1980s, and to identify recent demographic changes during the 12 year period studied, we estimated effective population size (N_e_) using the two-sample temporal method [43] developed by Jorde & Ryman [44] with the software NeESTIMATOR v2 [45]. We used the two-samples temporal method [45] because it is robust in the case of overlapping generations and can deal with low levels of polymorphisms [43, 44, 46], which are both concerns with this dataset.

We also evaluated the occurrence of recent bottlenecks for all samples singly and grouped by time point using the unilateral Wilcoxon test for heterozygosity excess implemented in the program BOTTLENECK v1.2.02 [47]. These tests were based on coalescence simulations at mutation-drift equilibrium under the two-phase model (TPM) and the infinite allele model (IAM), and used the mode-shift test [48] to detect deviations from the expected allele frequency distribution using the same program [47].

### Genetic differentiation among samples

To assess genetic structure among samples, we used the Bayesian clustering method implemented in STRUCTURE v2.3.4 [49]. This method uses a Markov Chain Monte Carlo (MCMC) procedure to group individuals into genetic clusters (K) that minimize deviation from HWE and reduce Linkage disequilibrium (LD) without a priori grouping. We allowed for admixture and inferred alpha in 10 independent runs for each K between 1 and 10, using a ‘burn-in’ period of 50,000 followed by 250,000 MCMC steps. We visualized results for each K value using the online software CLUMPAK [50], and visualized the estimated likelihoods of the data from each run using the online software STRUCTURE HARVESTER [51]. We also visualized patterns of variation among samples with a principal component analysis (PCA) using centroids and ellipses that encompassing 95% of the variance within each sample. We also estimated a neighbor-joining tree [52] using Nei’s genotype frequency-based distances [53] implemented in the “PopPR” v2.3.0 package [55, 56] in R with 1000 bootstrap replicates.

To assess levels of genetic differentiation among both geographically and temporally spaced samples, we estimated pairwise F_ST_ in Arlequin v.3.5 [42] with Wright’s statistics [56], following the variance method developed by Weir & Cockerham [57] and using 10,000 permutations to obtain exact *P*-values. With the resulting F-statistics converted to F_ST_/(1-F_ST_), we tested for isolation by distance, both geographically (km) and temporally (generations), using Rousset’s procedure [58] implemented in the “isolation by distance” v3.23 web service [59]. Geographical distances were generated using the web-based “geographic matrix generator” v1.2.3 [60], and the significance of the regression was tested by a Mantel test with 10,000 randomizations [61]. Temporal distances were calculated in number of generations assuming 2.4 months per generation (5 generations per year [62–64]). Temporal and geographical distances between samples are reported in Additional file 1: Table S4. To assess differences in allelic and genotypic frequencies among geographically and temporally spaced samples, we calculated the per locus frequencies and carried out Fisher’s exact tests between each sample [65] with 10,000 dememorizations, 1000 batches, and 10,000 iterations per batch, using Genepop [38]. Allele frequencies were visualized using 100% stacked bar charts in Microsoft Excel 2010.

## Results

### Microsatellite marker validation, genetic diversity, and demographic estimates

MICROCHECKER indicated null alleles in GpB6B and Gmm8. Results from tests for per-locus deviations from HWE and LD are presented in Additional file 1: Table S5 and Additional file 1: Table S6. There were significant HWE deviations in two or more samples in GpB6B and Gmm8 (Additional file 1: Table S5) and since these loci also had indications of null alleles, they were removed from the final dataset. There was significant LD in both regions between loci D05 and GpC10b after the Benjamini Hochberg [39] correction (Additional file 1: Table S6), so D05 was removed from the final dataset. Only the 11 loci with no evidence of null alleles and no significant deviations from expectations of neutral evolution and Mendelian inheritance (GpC5b, CmmK22, GmmC17, GmmL11, GmmA06, GpB20b, GpC10b, GpA19a, GpCAG133, GpC26b) were used in subsequent analyses.

Summary diversity statistics for all samples are presented in Table 1, and allele frequencies in each sample are plotted in Additional file 1: Figure S1. For Nguruman, per sample estimates of allelic richness ranged between 3.2–3.8 and averaged 3.8 (Table 1), and were 3.6 in the pooled sample from 2015, 3.9 in the pooled sample from 2009, and 3.4 in the pooled sample from 2003. Expected (H_E_) and observed (H_O_) heterozygosity was similar among replicates between sampling points, with H_O_ ranging between 0.406–0.504 (Table 1). F_IS_ estimates ranged bewteen -0.085–0.210 (Table 1) and were 0.033 in the pooled sample from 2015, 0.210 in the pooled sample from 2009, and -0.039 in the pooled sample from 2003, which indicates slight deviations from HWE expectations in 2003 and 2009. For Ruma, allelic richness ranged between 2.7–3.0 (Table 1), and were 3.7 in the pooled sample from 2015, 4.5 in the pooled sample from 2006, 4.0 in the pooled sample from 2005, and 3.9 in the pooled sample from 2003. H_E_ and H_O_ estimates were similar among replicates between the sampling points, with H_O_ ranging between 0.338–0.433 (Table 1). F_IS_ estimates ranged between 0.025–0.228 (Table 1), and were 0.117 in the pooled sample from 2015, -0.02 in the pooled sample from 2006, 0.127 in the pooled sample from 2005, and 0.013 in the pooled sample from 2003, again indicating slight deviations from HWE expectations.

**Table 1:** Summary statistics based on 13 microsatellite loci for *G. pallidipes* samples collected in Nguruman (LEN, MOK and PAK) by sample and pooled per year, and from Ruma (A, B and C) by sample and pooled per year

Locality	Latitude	Longitude	Month/Year	*n*	AR	H_O_	H_E_	F_IS_
Nguruman								
LEN-2015	1.9769	36.1167	February 2015	48	3.2	0.527	0.535	0.017
MOK-2015	-1.8316	36.0893	February 2015	53	3.7	0.499	0.525	0.049
PAK-2015	-1.9804	35.9825	February 2015	19	3.4	0.504	0.517	0.026
LEN-2009	-1.9582	36.1196	November 2009	29	3.8	0.406	0.511	0.210*
LEN-2003	-1.9576	36.1199	December 2003	30	3.2	0.534	0.493	-0.085
MOK-2003	-1.8246	36.0966	December 2003	59	3.4	0.535	0.523	-0.024
PAK-2003	-2.0000	36.0000	December 2003	29	3.2	0.524	0.508	-0.032
2015 (all sites)	na	na	February 2015	120	3.6	0.511	0.528	0.033
2009 (all sites)	na	na	November 2009	29	3.9	0.406	0.511	0.210*
2003 (all sites)	na	na	December 2003	118	3.4	0.533	0.513	-0.039*
Ruma								
RumaA-2015	-0.6082	34.3065	November 2015	30	2.8	0.355	0.401	0.117*
RumaA-2006	-0.6082	34.3065	October 2006	30	3.2	0.453	0.446	-0.015
RumaA-2005	-0.6082	34.3065	October 2005	10	2.7	0.422	0.425	0.007
RumaB-2005	-0.6531	34.2677	October 2005	10	2.8	0.398	0.474	0.168*
RumaC-2005	-0.7005	34.2200	October 2005	10	3.1	0.438	0.537	0.193*
RumaB-2003	-0.6531	34.2677	April 2003	30	3.1	0.446	0.452	0.013
2015	na	na	November 2015	30	3.7	0.355	0.401	0.117*
2006	na	na	October 2006	30	4.5	0.453	0.446	-0.015
2005	na	na	October 2005	30	4.0	0.362	0.414	0.127*
2003	na	na	April 2003	30	3.9	0.446	0.452	0.013

*Significant F_IS_ values indicating deviations from Hardy-Weinberg equilibrium (*P*-value <0.05)

*Abbreviations*: *n* number of samples analyzed, *na* not applicable, *AR* mean allelic richness among all loci, *H*_*O*_ observed heterozygosity, *H*_*E*_ expected heterozygosity, *F*_*IS*_ inbreeding coefficient

Tables 2 and 3 report the estimates of N_e_ and results from tests for bottlenecks, respectively, from pooled samples from each region and year. The single-sample estimates can be found in Additional file 1: Table S7. For the Nguruman, N_e_ estimates ranged between 144.0–731.7, with CIs ranging from an absolute low of 92.8 to an absolute high of 1067.2 (Table 2). BOTTLENECK results showed little indication of bottlenecks in any of the pooled samples (Table 3). Lenient tests under the IAM model showed a possible bottleneck in 2015 and 2003, but this was not supported by the analysis under the TPM model or by the mode-shift tests. For Ruma, N_e_ estimates [44] ranged between 170.0–1684.89, with CIs ranging from an absolute low of 101.8, to an absolute high of 2572 (Table 2). BOTTLENECK results showed no indication of bottlenecks under the IAM or TPM models, or with the mode-shift indicator test (Table 3). These results suggest no significant recent N_e_ reductions in Ruma.

**Table 2:** Results for samples pooled by year for estimates of effective population size (N_e_) based on the Jorde/Ryman temporal method showing the interval between samples in number of generations (Interval), the N_e_ estimate, the 95% confidence interval (CI)

Region	Collections used	Interval	N_e_	CI
Nguruman (all sites)	2003–2015	55.8	300.6	197.4–425.3
Nguruman (all sites)	2009–2015	26.3	144.0	92.8–206.3
Nguruman (all sites)	2003–2009	29.6	731.7	459–1067.2
Ruma (all sites)	2003–2015	62.9	697.5	403.3–1071.3
Ruma (all sites)	2005–2015	50.4	1684.8	983.6–2572.7
Ruma (all sites)	2006–2015	45.4	401.9	242.2–601.5
Ruma (all sites)	2003–2005	12.5	426.3	248.9–650.9
Ruma (all sites)	2003–2006	17.5	1465.3	876.8–∞
Ruma (all sites)	2005–2006	5.0	170.0	101.8–255.7

**Table 3:** Bottleneck test results bottleneck test results showing the *P*-values (*P)* under the two-phase model (TPM), the infinite allele model (IAM) and using the mode-shift test (a result of normal L-shaped (L) indicates no evidence of a bottleneck). All estimates of N_e_ were made using the software NeESTIMATOR v2 [50], and all tests for population bottlenecks were made using the software BOTTLENECK v. 1.2.02 [55]

Region	Collections used	TPM *P*	IAM *P*	Mode-Shift
Nguruman (all sites)	2015	0.500	0.009*	L
Nguruman (all sites)	2009	0.539	0.188	L
Nguruman (all sites)	2003	0.539	0.042*	L
Ruma (all sites)	2015	0.787	0.545	L
Ruma (all sites)	2006	0.947	0.722	L
Ruma (all sites)	2005	0.784	0.500	L
Ruma (all sites)	2003	0.820	0.326	L

*P < 0.05

### Genetic differentiation among samples

**Fig. 2:**
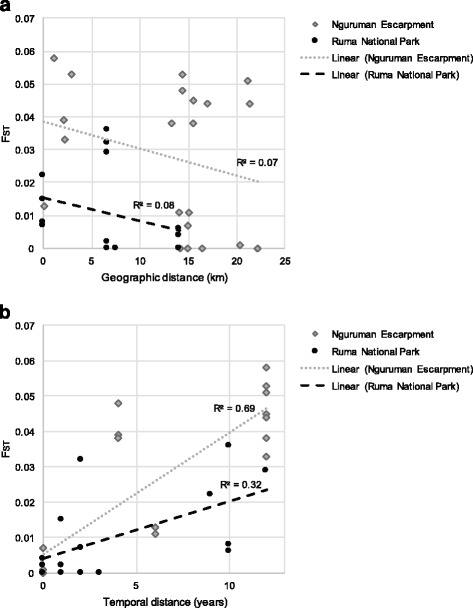
Results of tests for isolation by distance showing the linear regression of genetic differentiation F_ST_/(1-F_ST_) by geographical distance (km) (**a**), and temporal distance (generations) (**b**), with the best line of linear fit and R^2^ values displayed by region. Tests for isolation by distance were completed using Rousset’s procedure [63] implemented in the “isolation by distance” v3.23 web service [64]. Geographical distances were generated using the web-based “geographic matrix generator” v1.2.3 [65], and the significance of the regression (*P*) and strength of the correlation (*R* ^*2*^) were tested by a Mantel test with 10,000 randomizations [66]

Results indicate there was no differentiation among geographically spaced samples in either Nguruman or Ruma. This is suggested by the results of the Bayesian clustering analyses implemented using STRUCTURE (Additional file 1: Figure S2), and the PCA plots (Additional file 1: Figure S3), and by the NJ tree, which showed that samples from the same location do not form separate monophyletic groups Additional file 1: Figure S4. Lack of geographical structure is also supported by low F_ST_ estimates among geographically separated samples from the same years (Table 3), and no correlation between genetic and geographical distances (Nguruman: *R* ^*2*^ = 0.01, *P*-value = 0.748; Ruma: *R* ^*2*^ = 0.08, *P*-value = 0.820; Fig. 2a). Furthermore, there were no significant allelic/genotypic differences among geographically spaced samples from the same years (Table 6).

In contrast, there was strong evidence of genetic differentiation among temporally spaced samples in Nguruman but much weaker one among Ruma samples. The neighbor-joining tree grouped all 2015 samples together in 49.3% of bootstrap replicates and all 2003 samples together in 63.7% of bootstrap replicates (Additional file 1: Figure S4a). F_ST_ estimates ranged bewteen -0.003–0.059 (Table 3), with significant temporal differentiation between the 2015 samples and all other time points (Table 3). The Mantel test indicated significant correlation of genetic and temporal distance with a slope of 9.88e-4 (*R* ^*2*^ = 0.70, *P*-value = 0.013, Fig. 2b). Furthermore, Fisher’s exact tests comparing allelic and genotypic frequencies indicated that 13 out of 15 temporally spaced samples were significantly different from one another (Table 6), and none out of 6 geographically spaced samples were significantly different from one another (Table 6).

For Ruma, the neighbor joining tree did not cluster samples by year of collection (Additional file 1: Figure S4). F_ST_ estimates were low (Table 4), ranging between -0.017–0.036 (Table 4), with significant differentiation between 2003 and 2015 samples, some 2005 and 2006 samples, and 2003 and 2005 samples (Table 4). There was weak indication of differentiation among temporally spaced samples that did not create a significant pattern of isolation by temporal distance, as the Mantel tests indicated no correlation of genetic and temporal distances with a slope of 5.94e-4 (*R* ^*2*^ = 0.05, *P*-value = 0.277, Fig. 2b). Low levels of differentiation among temporal samples is also supported by the Fisher’s exact tests comparing allelic and genotypic frequencies.

**Table 4:** Pairwise F_ST_ values calculated in ARLEQUIN v.3.5 [47] with Wright’s statistics [56], following the variance method developed by Weir & Cockerham [57] and using 10,000 permutations to obtain exact *P*-values [61] from Nguruman

	LEN-2015	MOK-2015	PAK-2015	LEN-2009	LEN-2003	MOK-2003	PAK-2003
LEN-2015	–						
MOK-2015	0.001^a^	–					
PAK-2015	-0.003^a^	-0.001^a^	–				
LEN-2009	0.042*	0.045*	0.053*	–			
LEN-2003	0.042*	0.058*	0.059*	0.012	–		
MOK-2003	0.039*	0.050*	0.048*	0.008	-0.001^a^	–	
PAK-2003	0.041*	0.057*	0.048*	0.007	0.003^a^	-0.001^a^	–

*Significant *P*-values after Benjamini-Hochberg correction [44]

^a^Comparison of two samples from the same year

## Discussion

### Genetic diversity and demographic estimates

Allelic richness estimates indicate that Nguruman and Ruma exhibited levels of genetic diversity in samples pooled by year were similar to each other (ranging between 3.4–4.5; Table 1) and to those reported throughout the *G. pallidipes* distribution (ranging between 3.2–5.6 [25]). Allelic richness across both regions averaged 3.8 (Table 1), suggesting that genetic diversity was not substantially reduced by the control efforts during the 1960s to the1980s. This could be due to a variety of reasons that could have acted separately or together. It could be that the control measures were not maintained for long enough to impact the genetic diversity. Tsetse flies have relatively long life span compared to other insect vectors [66], and this could have allowed large enough numbers to survive in local relict populations, so that genetic diversity was maintained “on site”. This possibility is supported by positive F_IS_ estimates in 2009 in Nguruman, and in 2015 and 2005 in Ruma (Table 1). Positive F_IS_ indicates heterozygotes excess and is consistent with rapid demographic fluctuations and local geographical retractions and expansions [67]. Another possible explanation for the relatively high allelic richness could be recolonizations from neighboring regions after the control measures were discontinued. Both Nguruman and Ruma are geographically close to high tsetse densities in neighboring countries, making recolonizations from outside regions a real possibility. Indeed, a mix of individuals from different genetic backgrounds (i.e. the Wahlund effect) could also cause the finding of positive F_IS_ estimates (Table 1). Taken together, previous spatial analyses [16, 25] and the data presented here cannot distinguish between these possible scenarios.

N_e_ estimates were similar to those from other studies either on the same [20, 25] or a closely related species, *G. fuscipes fuscipes* [68–73]. Estimates comparable to other populations without a history of strong vector control suggest that the population reduction during the control efforts of the 1960s to the1980s either did not result in significant loss of genetic diversity or changes in allele frequencies, or that the populations rebounded quickly to pre-control effective population sizes. Future work with densely distributed markers such as Single Nucleotide Polymorphisms (SNPs) combined with the use of model based approaches to estimate demographic parameters, such as Approximate Bayesian Computation [74], could provide more precise N_e_ estimates.

Results from tests for bottlenecks do not provide strong evidence of significant genetic bottlenecks in either region. Although the methods used can only detect extreme reductions in population sizes that occurred more recently than 2–4 N_e_ generations ago [75, 76], they are sensitive enough to have detected genetic bottlenecks in *G. pallidipes* in previous studies [20, 75]. In tsetse flies, such as *G. pallidipes*, severe bottlenecks can probably be detected as far back as ~1400 generations, or 175 years, assuming N_e_ is close to the mean of ~700, as reported in other studies [68–73], and that the average number of generations is close to 5 per year [62–64]. Indead, none of the tests in samples grouped by year were significant in Ruma (Table 2), none of the tests using the most appropriate TPM model [47] were significant, and only two single time-point samples showed a signal of a bottleneck using the lenient IAM model (Table 3). Thus, indications of local bottlenecks are non-conclusive, and may simply reflect strong genetic drift in Nguruman.

### Genetic differentiation among samples

To evaluate the long-term population dynamics of *G. pallidipes*, we assessed levels and patterns of geographical and temporal genetic differentiation among samples from the two regions. We found no significant F_ST_ estimates or differnces in allelic or genotypic frequencies among geographically spaced samples collected during the same time interval in each region (Tables 4, 5), and no evidence of isolation by geographical distance (Fig. 2a). Although not unexpected, given the small geographical scale (Fig. 1) and levels of differentiation reported in other *G. pallidipes* studies [19], this result is different from the findings in the closely related species, *G. F. fuscipes*, where significant F_ST_ estimates were found at a spatial distance of just 5 km [54, 55]. The lack of geographical structure found in this study suggests either that *G. pallidipes* moves greater distances than *G. F. fuscipes*, or that the external environment in Nguruman and Ruma have facilitated gene flow in these regions.

**Table 5:** Pairwise F_ST_ values calculated in ARLEQUIN v.3.5 [47] with Wright’s statistics [56], following the variance method developed by Weir & Cockerham [57] and using 10,000 permutations to obtain exact *P*-values [61] from Ruma

	RumaA-2015	RumaA-2006	RumaA-2005	RumaB-2005	RumaC-2005	RumaB-2003
RumaA-2015	–					
RumaA-2006	0.029*	–				
RumaA-2005	0.006	0.028*	–			
RumaB-2005	0.034*	0.006	0.018^a^	–		
RumaC-2005	0.001	-0.010	0.017^a^	-0.017^a^	–	
RumaB-2003	0.022*	-0.003	0.036*	0.004	-0.009	–

*Significant *P*-values after Benjamini-Hochberg correction [44]

^a^Comparison of two samples from the same year

We identified significant temporal genetic differentiation in Nguruman, but not in Ruma. In Nguruman, we found significant genetic differentiation between samples collected before and after 2009 (Tables 4, 5; Additional file 1: Figure S4a) and a significant and tight correlation between genetic differentiation and temporal distance (Fig. 2b). These results are at odds with previous microsatellite based analyses in *G. pallidipes* by Ouma & Krafsur [16] and Ouma et al. [19], and in *G. fuscipes fuscipes* [56, 57], which did not detect genetic differentiation between temporal samples. The difference between these studies is probably due to a number of factors, one of which is the longer period covered in our study (12 years; ~96 generations) as compared to Ouma & Krafsur’s [18] and Ouma et al.’s [21] studies (3–12 months; ~2–8 generations), and Opiro et al.’s [56, 57] study (7 years,; ~56 generations). Another factor may be the timing of collections relative to population disruptions. For example, Ouma & Krafsur [18] focused on samples collected from Nguruman in 2001–2003 and did not find any differentiation, while our samples from the same locality from 2003 to 2015 showed significant differentiation. These findings indicate that temporal genetic differentiation occurs in *G. pallidipes* and can be detected at a scale of > 80 generations under some conditions. These findings also suggest that previous studies could not detect temporal differnetioan in Nguruman [16] because samples were collected too close in time to each other, and thus, future population genetic schemes should include a broad time frame to better detect temporal genetic differentiaion in tsetse.

### Implications for vector control

Genetic diversity, N_e_, and tests for bottlenecks indicate a similar response to intensive control efforts between the 1960s and the1980s between Nguruman and Ruma. There was no evidence of significantly reduce genetic diversity, and estimates were similar to those reported in other regions without a history of intensive control measures [20, 25]. Furthermore, results from the bottleneck tests did not indicate genetic bottlencks. These findings suggest that for *G. pallidipes*, even massive control efforts have failed to have a long-term influence on genetic diversity and allele frequency. This could be because control measures were not maintained for long enough to impact the genetic diversity, or because there were recolonizations from neighboring regions after the control measures were discontinued.

However, we did find differences in the results of the bottleneck tests and levels of temporal genetic differentiation between these two regions. Nguruman displayed higher levels of differentiation (Fig. 2; Additional file 1: Fig. S4) and larger changes in allele/genotype frequencies between temporally spaced samples (Table 6). Differences could suggest that populations in these regions responded to the control campaign and its halting in different ways, or that populations in these regions were exposed to different conditions after the control measures were halted. The most obvious difference between the regions is that Ruma is protected from human use, while Nguruman is not. This different status has contributed to land-use alterations in Nguruman, including construction of irrigation systems and deforestation, as land was converted for cattle grazing and agriculture [26–29]. In addition,Nguruman has also been heavily impacted by Maasai animal grazing and trade, with cattle being brought in from as far as Lake Natron in Northern Tanzania [77, 78]. It is possible that these human activities may have disrupted the *G. pallidipes* habitats in Nguruman in ways that did not occur in Ruma, where land is fully protected from human use. We suggest that alteration of tsetse habitats due to human use [26–29] may be one of the main drivers for the temporal genetic changes detected in Nguruman. Another possibility that cannot be assessed with this dataset was continuous low levels of gene flow from outside areas druing the period studied that drove changes in allele and genotype frequencies observed. An important next step in understanding the drivers of temporal differentiation in Ngurman will be to assess genetic variation within a larger geographical context and with more advanced statistical approaches. For example, methods such as isolation by resistance [79, 80] and by environment [81] can help assess the impact of land use transformation on genetic data, by providing a way to disentangle the effect of geographical and ecological differentiation on genetic differentiation [82].

**Table 6:** Fisher’s exact test for genotypic and allelic differentiation of 13 microsatellites from Nguruman and Ruma, organized by locality and year of collection, showing the comparison, the sample pair used in the test

Comparison	Sample pair	Genotypic			Allelic		
		*χ* ^2^	*df*	*P*-value	*χ* ^2^	*df*	*P*-value
Nguruman							
Within LEN	LEN-2015 & LEN-2009	∞	20	< 0.001*	∞	20	< 0.001*
	LEN-2015 & LEN-2003	∞	20	< 0.001*	∞	20	< 0.001*
	LEN-2009 & LEN-2003	27.2	20	0.131	23.0	20	0.287
LEN & MOK	LEN-2015 & MOK-2015^a^	30.0	20	0.070	26.8	20	0.130
	LEN-2015 & MOK-2003	∞	20	< 0.001*	∞	20	< 0.001*
	LEN-2009 & MOK-2015	∞	20	< 0.001*	∞	20	< 0.001*
	LEN-2009 & MOK-2003	34.6	20	0.022	32.5	20	0.038
	LEN-2003 & MOK-2015	∞	20	< 0.001*	∞	20	< 0.001*
	LEN-2003 & MOK-2003^a^	18.3	20	0.569	18.7	20	0.540
LEN & PAK	LEN-2015 & PAK-2015^a^	13.7	20	0.847	13.1	20	0.872
	LEN-2015 & PAK-2003	∞	20	< 0.001*	∞	20	< 0.001*
	LEN-2009 & PAK-2003	∞	20	< 0.001*	∞	20	< 0.001*
	LEN-2009 & PAK-2015	23.3	20	0.272	22.1	20	0.334
	LEN-2003 & PAK-2015	∞	20	< 0.001*	∞	20	< 0.001*
	LEN-2003 & PAK-2003^a^	18.5	20	0.556	20.1	20	0.448
Within MOK	MOK-2015 & MOK-2003	∞	20	< 0.001*	∞	20	< 0.001*
MOK & PAK	MOK-2015 & PAK-2015^a^	14.2	20	0.818	16.3	20	0.701
	MOK-2015 & PAK-2003	∞	20	< 0.001*	∞	20	< 0.001*
	MOK-2003 & PAK-2015	∞	20	< 0.001*	∞	20	< 0.001*
	MOK-2003 & PAK-2003^a^	21.2	20	0.383	22.6	20	0.307
Within PAK	PAK-2015 & PAK-2003	∞	20	< 0.001*	∞	20	< 0.001*
Ruma							
within Block A	RumaA-2015 & RumaA-2006	43.6	20	0.002*	37.2	20	0.011*
	RumaA-2015 & RumaA-2005	22.2	20	0.329	26.1	20	0.164
	RumaA-2006 & RumaA-2005	20.6	20	0.418	18.6	20	0.547
Block A & B	RumaA-2015 & RumaB-2005	∞	18	< 0.001*	∞	18	< 0.001*
	RumaA-2015 & RumaB-2003	37.8	18	0.004*	34.0	18	0.012*
	RumaA-2006 & RumaB-2005	12.0	20	0.918	9.8	20	0.971
	RumaA-2006 & RumaB-2003	14.7	20	0.796	14.5	20	0.803
	RumaA-2005 & RumaB-2005^a^	22.2	20	0.331	18.9	20	0.529
	RumaA-2005 & RumaB-2003	25.3	20	0.188	27.5	20	0.121
Block A & C	RumaA-2015 & RumaC-2005	25.5	18	0.111	22.2	18	0.221
	RumaA-2006 & RumaC-2005	12.0	20	0.915	13.3	20	0.862
	RumaA-2005 & RumaC-2005^a^	19.0	20	0.519	15.2	20	0.767
within Block B	RumaB-2005 & RumaB-2003	14.1	18	0.722	15.2	18	0.649
Block B & C	RumaB-2005 & RumaC-2005^a^	8.7	18	0.966	7.7	18	0.983
	RumaB-2003 & RumaC-2005	12.0	18	0.847	12.8	18	0.802

^a^Comparison of two samples from the same year

*P < 0.05

## Conclusions

Our findings suggest that for *G. pallidipes,* even massive control efforts have not had lasting influence on genetic diversity, and that temporal genetic differentiation occurs in *G. pallidipes* under some circumstances, such as human land use. These findings are important because they enhance our understanding of the process underlying the local persistence of *G. pallidipes* populations in southwestern Kenya over the past four decades, stress the importance of long-term monitoring and sustained control efforts in control of *G. pallidipes* in southwestern Kenya, and suggest that different habitat management can play a significant role in shaping levels of genetic differentiation in this vector. In addition, they also point out important knowledge gaps which can be addressed by future research, such as the need of densely spaced genomic markers and of a sampling design that includes both target and neighboring populations. This would facilitate estimates of key demographic parameters and reconstructions of recent and past gene flow levels and patterns among different sampling sites. Improved estimates will increase our ability to identify the ecological, evolutionary, and/or human-mediated process underlying the genetic changes between temporally spaced *G. pallidipes* samples.

## Additonal file

Additional file 1: Table S1. Microsatellite locus name, dye used, primer sequences, size range of the amplified product, repeat motif length and the original publication sourced for primer design. **Table S2.** Microsatellite multiplex name, and the locus marked with each dye. **Table S3.** GeneMarker panel binning rules, including multiplex design, upper and lower size (bp) boundaries for each marker, allele names, and binning center, minimum, and maximum. **Table S4.** Pairwise geographical distance (km) and temporal distance (years) between samples from (**a**) Nguruman, and (**b**) Ruma. **Table S5.** Per locus F_IS_ values obtained after testing for deviation from Hardy-Weinberg equilibrium (HWE) using FSTAT v2.9.3 [46] based on 10,000 randomizations for (**a**) Nguruman and (**b**) Ruma. **Table S6.** Pairwise locus *P*-values after testing for linkage disequilibrium estimated in Genepop v4.1 [43]. **Table S7.** Per-sample (**a**) estimates of effective population size (N_e_) based on the Jorde/Ryman temporal method showing the interval used in generations (I), the N_e_ estimate, the 95% CI, and (**b**) bottleneck results showing the *P*-values (*P)* under the two-phase model (TPM), the infinite allele model (IAM) and using the mode-shift test. **Figure S1**. Allele frequency of each of the 10 microsatellites from (**a**) Nguruman and (**b**) Ruma. **Figure S2**. STRUCTURE [54] estimated Ln likelihood of the data for K values 1–10 for (**a**) Nguruman and (**b**) Ruma, and plots of structure assignment for K values 2–10 for (**c**) Nguruman and (**d**) Ruma. **Figure S3.** Principal Components Analysis (PCA) of microsatellite data from (**a**) Nguruman and (**b**) Ruma. **Figure S4.** Neighbor-joining tree [57] using Nei’s genotype frequency based distances (**a**) Nguruman and (**b**) Ruma. (DOCX 1088 kb)

## Abbreviations

AAT: Animal African Trypanosomiasis
BSA: Bovine serum albumin
DAPC: Discriminant Analysis of Principal Components
dNTP: Deoxynucleotide triphosphate
F_CT_: Variance among groups relative to the total variance
F_IS_: Inbreeding coefficient
F_IT_: Variance within individuals
F_SC_: Variance among subpopulations within groups
F_ST_: Variance among subpopulations relative to the total variance
HAT: Human African Trypanosomiasis
H_e_: Expected heterozygosity
H_o_: Observed heterozygosity
HWE: Hardy-Weinberg equilibrium
IAEA: International Atomic Energy Agency
LEN: Lengong
MOK: Mukinyo
N_e_: Effective population size
PAK: Pakase
TPM: Two-phase model
WHO: World Health Organization
