# Supplementary material for: Temporal genetic differentiation in Glossina pallidipes tsetse fly populations in Kenya
Source: Parasit Vectors. 2017 Oct 10;10:471. doi: 10.1186/s13071-017-2415-y (PMC5635580; doi:10.1186/s13071-017-2415-y)
Supplement: Additional file 1: Table S1. — Microsatellite locus name, dye used, primer sequences, size range of the amplified product, repeat motif length and the original publication sourced for primer design. Table S2. Microsatellite multiplex name, and the locus marked with each dye. Table S3. GeneMarker panel binning rules, including multiplex design, upper and lower size (bp) boundaries for each marker, allele names, and binning center, minimum, and maximum. Table S4. Pairwise geographical distance (km) and temporal distance (years) between samples from (a) Nguruman, and (b) Ruma. Table S5. Per locus FIS values obtained after testing for deviation from Hardy-Weinberg equilibrium (HWE) using FSTAT v2.9.3 [46] based on 10,000 randomizations for (a) Nguruman and (b) Ruma. Table S6. Pairwise locus P-values after testing for linkage disequilibrium estimated in Genepop v4.1 [43]. Table S7. Per-sample (a) estimates of effective population size (Ne) based on the Jorde/Ryman temporal method showing the interval used in generations (I), the Ne estimate, the 95% CI, and (b) bottleneck results showing the P-values (P) under the two-phase model (TPM), the infinite allele model (IAM) and using the mode-shift test. Figure S1. Allele frequency of each of the 10 microsatellites from (a) Nguruman and (b) Ruma. Figure S2. STRUCTURE [54] estimated Ln likelihood of the data for K values 1–10 for (a) Nguruman and (b) Ruma, and plots of structure assignment for K values 2–10 for (c) Nguruman and (d) Ruma. Figure S3. Principal Components Analysis (PCA) of microsatellite data from (a) Nguruman and (b) Ruma. Figure S4. Neighbor-joining tree [57] using Nei’s genotype frequency based distances (a) Nguruman and (b) Ruma. (DOCX 1088 kb) [file 13071_2017_2415_MOESM1_ESM.docx]

**Additional File 1**

**Table S1.** Microsatellite locus name, dye used, primer sequences, size range of the amplified product, repeat motif length and the original publication sourced for primer design.

| **Locus** | **Dye** | **F Primer** | **R Primer** | **Size Range** | **Repeat Motif Length** | **Source** |
| --- | --- | --- | --- | --- | --- | --- |
| GmmK22 | TAM | ACGCTTACGTTTCCGTTACAC | AAGCTAACCGAACCAGCAC | 192-198 | 3 | [35,38] |
| GmmA06 | TAM | ACTTCCATGTTATGTTCGTTGC | TGCCTTAGTTGAGAAACTCTGC | 154-166 | 2 | [35,38] |
| GmmC17 | HEX | TGCGCTTTGAACGGAACG | CTATGCCGCCTGGCTTATC | 190-202 | 4 | [35,38] |
| GmmL11 | HEX | CCACCACTAACAACGACAGC | TGGCTGGTTACAAGATTGCAC | 250-252 | 2 | [35,38] |
| GpB20b | FAM | AGTTTGCTTCTCAACGCAGTAG | GTTTCGGCAGTAGATGGCAA | 139-200 | 2 | [36] |
| GpA19a | FAM | CATATCCACACCCACATACAT | GTTTGCGATTATGGCTAGAGGTTT | 142-189 | 2 | [36] |
| GpCAG133 | HEX | ATTTTTGCGTCAACGTGA | GTTTATGAGGATGTTGTCCAGTTT | 185-209 | 3 | [36] |
| GpC5b | NED | GTTGTTTTCTGCTCCTCAATA | GTTTCAAGGGTGTGTCGTCTTC | 187-239 | 3 | [36] |
| GpC10b | NED | GTTGATGTTGTGATGGTAATGA | GTTTGCTGGCAAAGAAACTAATGA | 283-314 | 3 | [36] |
| GpC26b | FAM | GGATCACCCTTCTTGAATG | GTTTGGACGTTATTTGTTCGTGTAA | 168-201 | 3 | [36] |

**Table S2.** Microsatellite multiplex name, and the locus marked with each dye.

| **Multiplex name** | **Loci marked**  **with FAM** | **Loci marked with HEX** | **Loci marked with TAM/NED** |
| --- | --- | --- | --- |
| Gp_I | n/a | GmmC17 | GmmK22 |
| Gp_II | n/a | GpCAG133 | n/a |
| Gp_III | n/a | GmmL11 | GmmA06 |
| Gp_IV | n/a | n/a | GpC5b |
| Gp_V | GpA19a | n/a | GpC10b |
| Gp_VI | GpB20b | n/a | n/a |
| Gp_VII | GpC26b | n/a | n/a |

**Table S3.** GeneMarker panel binning rules, including multiplex design, upper and lower size (bp) boundaries for each marker, allele names, and binning center, minimum, and maximum.

|  | **Locus** | **Upper**  **limit** | **Lower**  **limit** | **Allele Name** | **Center** | **Start** | **Stop** |
| --- | --- | --- | --- | --- | --- | --- | --- |
| **Multiplex 1** | |  |  |  |  |  |  |
|  | **D05** | 122.1 | 93.0 | 94 | 94.00 | 93.00 | 95.00 |
|  |  |  |  | 97 | 97.00 | 96.00 | 98.00 |
|  |  |  |  | 100 | 100.00 | 99.00 | 101.00 |
|  |  |  |  | 103 | 103.00 | 102.00 | 104.00 |
|  |  |  |  | 106 | 106.00 | 105.00 | 107.00 |
|  |  |  |  | 109 | 109.00 | 108.00 | 110.00 |
|  |  |  |  | 112 | 112.00 | 111.00 | 113.00 |
|  |  |  |  | 115 | 115.00 | 114.00 | 116.00 |
|  |  |  |  | 118 | 118.00 | 117.00 | 119.00 |
|  |  |  |  | 121 | 121.00 | 120.00 | 122.00 |
|  | **GmmC17** | 205.9 | 167.3 | 170 | 168.42 | 167.42 | 169.42 |
|  |  |  |  | 174 | 174.94 | 173.94 | 175.94 |
|  |  |  |  | 178 | 177.09 | 176.09 | 178.09 |
|  |  |  |  | 182 | 181.43 | 180.43 | 182.43 |
|  |  |  |  | 186 | 186.20 | 185.20 | 187.20 |
|  |  |  |  | 190 | 190.20 | 189.20 | 191.20 |
|  |  |  |  | 194 | 192.30 | 191.30 | 193.30 |
|  |  |  |  | 198 | 196.60 | 195.60 | 197.60 |
|  |  |  |  | 202 | 202.10 | 201.10 | 203.10 |
|  |  |  |  | 206 | 204.80 | 203.80 | 205.80 |
|  | **GmK22** | 206.6 | 188.4 | 191 | 189.50 | 188.50 | 190.50 |
|  |  |  |  | 194 | 193.50 | 192.50 | 194.50 |
|  |  |  |  | 197 | 196.60 | 195.60 | 197.60 |
|  |  |  |  | 200 | 199.80 | 198.80 | 200.80 |
|  |  |  |  | 203 | 202.80 | 201.80 | 203.80 |
|  |  |  |  | 206 | 205.45 | 204.45 | 206.45 |
| **Multiplex 2** | |  |  |  |  |  |  |
|  | **GpCAG133** | 198.2 | 171.9 | 173 | 173.00 | 172.00 | 174.00 |
|  |  |  |  | 176 | 176.10 | 175.10 | 177.10 |
|  |  |  |  | 179 | 178.80 | 177.80 | 179.80 |
|  |  |  |  | 182 | 182.20 | 181.20 | 183.20 |
|  |  |  |  | 185 | 185.10 | 184.10 | 186.10 |
|  |  |  |  | 188 | 188.10 | 187.10 | 189.10 |
|  |  |  |  | 191 | 191.10 | 190.10 | 192.10 |
|  |  |  |  | 194 | 194.10 | 193.10 | 195.10 |
|  |  |  |  | 197 | 197.10 | 196.10 | 198.10 |
| **Multiplex 3** | |  |  |  |  |  |  |
|  | **GmL11** | 269.6 | 206.9 | 208 | 208.00 | 207.00 | 209.00 |
|  |  |  |  | 210 | 210.00 | 209.00 | 211.00 |
|  |  |  |  | 212 | 212.00 | 211.00 | 213.00 |
|  |  |  |  | 214 | 214.00 | 213.00 | 215.00 |
|  |  |  |  | 216 | 216.00 | 215.00 | 217.00 |
|  |  |  |  | 218 | 218.00 | 217.00 | 219.00 |
|  |  |  |  | 220 | 220.00 | 219.00 | 221.00 |
|  |  |  |  | 222 | 222.00 | 221.00 | 223.00 |
|  |  |  |  | 224 | 224.00 | 223.00 | 225.00 |
|  |  |  |  | 226 | 226.00 | 225.00 | 227.00 |
|  |  |  |  | 228 | 228.00 | 227.00 | 229.00 |
|  |  |  |  | 230 | 230.00 | 229.00 | 231.00 |
|  |  |  |  | 232 | 232.00 | 231.00 | 233.00 |
|  |  |  |  | 234 | 234.00 | 233.00 | 235.00 |
|  |  |  |  | 236 | 236.00 | 235.00 | 237.00 |
|  |  |  |  | 238 | 238.00 | 237.00 | 239.00 |
|  |  |  |  | 240 | 240.00 | 239.00 | 241.00 |
|  |  |  |  | 242 | 242.00 | 241.00 | 243.00 |
|  |  |  |  | 244 | 244.00 | 243.00 | 245.00 |
|  |  |  |  | 246 | 246.00 | 245.00 | 247.00 |
|  |  |  |  | 248 | 248.00 | 247.00 | 249.00 |
|  |  |  |  | 250 | 250.00 | 249.00 | 251.00 |
|  |  |  |  | 252 | 252.00 | 251.00 | 253.00 |
|  |  |  |  | 254 | 254.00 | 253.00 | 255.00 |
|  |  |  |  | 256 | 256.00 | 255.00 | 257.00 |
|  |  |  |  | 258 | 258.50 | 257.50 | 259.50 |
|  |  |  |  | 260 | 260.50 | 259.50 | 261.50 |
|  |  |  |  | 262 | 262.50 | 261.50 | 263.50 |
|  |  |  |  | 264 | 264.50 | 263.50 | 265.50 |
|  |  |  |  | 266 | 266.50 | 265.50 | 267.50 |
|  |  |  |  | 268 | 268.50 | 267.50 | 269.50 |
|  | **GmA06** | 179.1 | 138.9 | 140 | 140.00 | 139.00 | 141.00 |
|  |  |  |  | 142 | 142.00 | 141.00 | 143.00 |
|  |  |  |  | 144 | 144.00 | 143.00 | 145.00 |
|  |  |  |  | 146 | 146.00 | 145.00 | 147.00 |
|  |  |  |  | 148 | 148.00 | 147.00 | 149.00 |
|  |  |  |  | 150 | 150.00 | 149.00 | 151.00 |
|  |  |  |  | 152 | 152.00 | 151.00 | 153.00 |
|  |  |  |  | 154 | 154.00 | 153.00 | 155.00 |
|  |  |  |  | 156 | 156.00 | 155.00 | 157.00 |
|  |  |  |  | 158 | 158.00 | 157.00 | 159.00 |
|  |  |  |  | 160 | 160.00 | 159.00 | 161.00 |
|  |  |  |  | 162 | 162.00 | 161.00 | 163.00 |
|  |  |  |  | 164 | 164.00 | 163.00 | 165.00 |
|  |  |  |  | 166 | 166.00 | 165.00 | 167.00 |
|  |  |  |  | 168 | 168.00 | 167.00 | 169.00 |
|  |  |  |  | 170 | 170.00 | 169.00 | 171.00 |
|  |  |  |  | 172 | 172.00 | 171.00 | 173.00 |
|  |  |  |  | 174 | 174.00 | 173.00 | 175.00 |
|  |  |  |  | 176 | 176.00 | 175.00 | 177.00 |
|  |  |  |  | 178 | 178.00 | 177.00 | 179.00 |
| **Multiplex 4** | |  |  |  |  |  |  |
|  | **GpC5b** | 235.1 | 208.9 | 210 | 210.00 | 209.00 | 211.00 |
|  |  |  |  | 213 | 213.00 | 212.00 | 214.00 |
|  |  |  |  | 216 | 216.00 | 215.00 | 217.00 |
|  |  |  |  | 219 | 219.00 | 218.00 | 220.00 |
|  |  |  |  | 222 | 222.00 | 221.00 | 223.00 |
|  |  |  |  | 225 | 225.00 | 224.00 | 226.00 |
|  |  |  |  | 228 | 228.00 | 227.00 | 229.00 |
|  |  |  |  | 231 | 231.00 | 230.00 | 232.00 |
|  |  |  |  | 234 | 234.00 | 233.00 | 235.00 |
| **Multiplex 5** | |  |  |  |  |  |  |
|  | **GpA19a** | 164.1 | 133.9 | 135 | 135.00 | 134.00 | 136.00 |
|  |  |  |  | 137 | 137.00 | 136.00 | 138.00 |
|  |  |  |  | 139 | 139.00 | 138.00 | 140.00 |
|  |  |  |  | 141 | 141.00 | 140.00 | 142.00 |
|  |  |  |  | 143 | 143.00 | 142.00 | 144.00 |
|  |  |  |  | 145 | 145.00 | 144.00 | 146.00 |
|  |  |  |  | 147 | 147.00 | 146.00 | 148.00 |
|  |  |  |  | 149 | 149.00 | 148.00 | 150.00 |
|  |  |  |  | 151 | 151.00 | 150.00 | 152.00 |
|  |  |  |  | 153 | 153.00 | 152.00 | 154.00 |
|  |  |  |  | 155 | 155.00 | 154.00 | 156.00 |
|  |  |  |  | 157 | 157.00 | 156.00 | 158.00 |
|  |  |  |  | 159 | 159.00 | 158.00 | 160.00 |
|  |  |  |  | 161 | 161.00 | 160.00 | 162.00 |
|  |  |  |  | 163 | 163.00 | 162.00 | 164.00 |
|  | **GpC10b** | 316.1 | 277.9 | 279 | 279.00 | 278.00 | 280.00 |
|  |  |  |  | 282 | 282.00 | 281.00 | 283.00 |
|  |  |  |  | 285 | 285.00 | 284.00 | 286.00 |
|  |  |  |  | 288 | 288.00 | 287.00 | 289.00 |
|  |  |  |  | 291 | 291.00 | 290.00 | 292.00 |
|  |  |  |  | 294 | 294.00 | 293.00 | 295.00 |
|  |  |  |  | 297 | 297.00 | 296.00 | 298.00 |
|  |  |  |  | 300 | 300.00 | 299.00 | 301.00 |
|  |  |  |  | 303 | 303.00 | 302.00 | 304.00 |
|  |  |  |  | 306 | 306.00 | 305.00 | 307.00 |
|  |  |  |  | 309 | 309.00 | 308.00 | 310.00 |
|  |  |  |  | 312 | 312.00 | 311.00 | 313.00 |
|  |  |  |  | 315 | 315.00 | 314.00 | 316.00 |
| **Multiplex 6** | |  |  |  |  |  |  |
|  | **GpB20b** | 200.1 | 135.9 | 137 | 137.00 | 136.00 | 138.00 |
|  |  |  |  | 139 | 139.00 | 138.00 | 140.00 |
|  |  |  |  | 141 | 141.00 | 140.00 | 142.00 |
|  |  |  |  | 143 | 143.00 | 142.00 | 144.00 |
|  |  |  |  | 145 | 145.00 | 144.00 | 146.00 |
|  |  |  |  | 147 | 147.00 | 146.00 | 148.00 |
|  |  |  |  | 149 | 149.00 | 148.00 | 150.00 |
|  |  |  |  | 151 | 151.00 | 150.00 | 152.00 |
|  |  |  |  | 153 | 153.00 | 152.00 | 154.00 |
|  |  |  |  | 155 | 155.00 | 154.00 | 156.00 |
|  |  |  |  | 157 | 157.00 | 156.00 | 158.00 |
|  |  |  |  | 159 | 159.00 | 158.00 | 160.00 |
|  |  |  |  | 161 | 161.00 | 160.00 | 162.00 |
|  |  |  |  | 163 | 163.00 | 162.00 | 164.00 |
|  |  |  |  | 165 | 165.00 | 164.00 | 166.00 |
|  |  |  |  | 167 | 167.00 | 166.00 | 168.00 |
|  |  |  |  | 169 | 169.00 | 168.00 | 170.00 |
|  |  |  |  | 171 | 171.00 | 170.00 | 172.00 |
|  |  |  |  | 173 | 173.00 | 172.00 | 174.00 |
|  |  |  |  | 175 | 175.00 | 174.00 | 176.00 |
|  |  |  |  | 177 | 177.00 | 176.00 | 178.00 |
|  |  |  |  | 179 | 179.00 | 178.00 | 180.00 |
|  |  |  |  | 181 | 181.00 | 180.00 | 182.00 |
|  |  |  |  | 183 | 183.00 | 182.00 | 184.00 |
|  |  |  |  | 185 | 185.00 | 184.00 | 186.00 |
|  |  |  |  | 187 | 187.00 | 186.00 | 188.00 |
|  |  |  |  | 189 | 189.00 | 188.00 | 190.00 |
|  |  |  |  | 191 | 191.00 | 190.00 | 192.00 |
|  |  |  |  | 193 | 193.00 | 192.00 | 194.00 |
|  |  |  |  | 195 | 195.00 | 194.00 | 196.00 |
|  |  |  |  | 197 | 197.00 | 196.00 | 198.00 |
|  |  |  |  | 199 | 199.00 | 198.00 | 200.00 |
| **Multiplex 7** | |  |  |  |  |  |  |
|  | **GpC26b** | 209.1 | 170.9 | 172 | 172.00 | 171.00 | 173.00 |
|  |  |  |  | 175 | 175.00 | 174.00 | 176.00 |
|  |  |  |  | 178 | 178.00 | 177.00 | 179.00 |
|  |  |  |  | 181 | 181.00 | 180.00 | 182.00 |
|  |  |  |  | 184 | 184.00 | 183.00 | 185.00 |
|  |  |  |  | 187 | 187.00 | 186.00 | 188.00 |
|  |  |  |  | 190 | 190.00 | 189.00 | 191.00 |
|  |  |  |  | 193 | 193.00 | 192.00 | 194.00 |
|  |  |  |  | 196 | 196.00 | 195.00 | 197.00 |
|  |  |  |  | 199 | 199.00 | 198.00 | 200.00 |
|  |  |  |  | 202 | 202.00 | 201.00 | 203.00 |
|  |  |  |  | 205 | 205.00 | 204.00 | 206.00 |
|  |  |  |  | 208 | 208.00 | 207.00 | 209.00 |

**Table S4.** Pairwise geographical distance (km) and temporal distance (years) between samples from (**a**) Nguruman, and **(b)** Ruma.

| **(a)** | | | | |
| --- | --- | --- | --- | --- |
| **Sample 1** | **Sample 2** | **Temporal distance (months)** | **Temporal distance (generations)** | **Geographic distance (km)** |
| LEN-2015 | LEN-2009 | 63 | 26.3 | 2.09 |
| LEN-2015 | LEN-2003 | 144 | 55.8 | 2.16 |
| LEN-2015 | PAK-2015 | 0 | 0.0 | 16.35 |
| LEN-2015 | PAK-2003 | 144 | 55.8 | 16.99 |
| LEN-2015 | MOK-2015 | 0 | 0.0 | 14.94 |
| LEN-2015 | MOK-2003 | 144 | 55.8 | 13.23 |
| LEN-2009 | LEN-2003 | 71 | 29.6 | 0.07 |
| LEN-2009 | PAK-2015 | 63 | 26.3 | 14.4 |
| LEN-2009 | PAK-2003 | 71 | 29.6 | 14.99 |
| LEN-2009 | MOK-2015 | 63 | 26.3 | 15.45 |
| LEN-2009 | MOK-2003 | 71 | 29.6 | 14.09 |
| LEN-2003 | PAK-2015 | 144 | 55.8 | 14.34 |
| LEN-2003 | PAK-2003 | 0 | 0.0 | 14.93 |
| LEN-2003 | MOK-2015 | 144 | 55.8 | 15.49 |
| LEN-2003 | MOK-2003 | 0 | 0.0 | 14.14 |
| PAK-2015 | PAK-2003 | 144 | 55.8 | 1.12 |
| PAK-2015 | MOK-2015 | 0 | 0.0 | 20.3 |
| PAK-2015 | MOK-2003 | 144 | 55.8 | 21.11 |
| PAK-2003 | MOK-2015 | 144 | 55.8 | 21.4 |
| PAK-2003 | MOK-2003 | 0 | 0.0 | 22.17 |
| MOK-2015 | MOK-2003 | 144 | 55.8 | 2.91 |

| **(b)** | | | | |
| --- | --- | --- | --- | --- |
| **Sample 1** | **Sample 2** | **Temporal distance (months)** | **Temporal distance (generations)** | **Geographic distance (km)** |
| RumaA-2015 | RumaA-2006 | 109 | 45.4 | 0 |
| RumaA-2015 | RumaA-2005 | 109 | 45.4 | 0 |
| RumaA-2015 | RumaB-2005 | 121 | 50.4 | 6.58 |
| RumaA-2015 | RumaB-2003 | 121 | 50.4 | 6.58 |
| RumaA-2015 | RumaC-2005 | 121 | 50.4 | 14.03 |
| RumaA-2006 | RumaA-2005 | 109 | 45.4 | 0 |
| RumaA-2006 | RumaB-2005 | 121 | 50.4 | 6.58 |
| RumaA-2006 | RumaB-2003 | 121 | 50.4 | 6.58 |
| RumaA-2006 | RumaC-2005 | 121 | 50.4 | 14.03 |
| RumaA-2005 | RumaB-2005 | 121 | 50.4 | 6.58 |
| RumaA-2005 | RumaB-2003 | 121 | 50.4 | 6.58 |
| RumaA-2005 | RumaC-2005 | 121 | 50.4 | 14.03 |
| RumaB-2005 | RumaB-2003 | 109 | 45.4 | 0 |
| RumaB-2005 | RumaC-2005 | 0 | 0.0 | 7.46 |
| RumaB-2003 | RumaC-2005 | 0 | 0.0 | 7.46 |

**Table S5.** Per locus F_IS_ values obtained after testing for deviation from Hardy-Weinberg Equilibrium (HWE) using FSTAT v2.9.3 [46] based on 10,000 randomizations for **(a)** Nguruman and **(b)** Ruma.

| **(a)** | | | | | | | |
| --- | --- | --- | --- | --- | --- | --- | --- |
| **Locus** | **LEN-2015** | **MOK-2015** | **PAK-2015** | **LEN-2009** | **MOK-2003** | **PAK-2003** | **LEN-2003** |
| GpC5b | -0.108 | -0.038 | 0.236 | 0.312 | -0.045 | -0.008 | -0.182 |
| GmmK22 | 0.135 | 0.062 | -0.241 | 0.183 | **-0.459** | -0.386 | -0.308 |
| GmmC17 | 0.134 | 0.176 | 0.077 | 0.225 | **0.126** | 0.028 | 0.075 |
| GmmL11 | 0.048 | 0.025 | -0.104 | -0.0133 | 0.215 | -0.044 | -0.050 |
| GmmA06 | 0.016 | 0.056 | -0.304 | 0.319 | -0.131 | -0.092 | 0.087 |
| GpB20b | -0.213 | -0.054 | 0.325 | 0.213 | 0.166 | 0.153 | -0.082 |
| GpC10b | 0.191 | 0.103 | 0.157 | 0.253 | -0.013 | -0.078 | -0.077 |
| GpA19a | -0.033 | 0.075 | 0.202 | 0.067 | 0.081 | -0.055 | 0.016 |
| GpCAG133 | 0.066 | 0.016 | 0.180 | 0.233 | -0.028 | 0.103 | -0.208 |
| GpC26b | -0.041 | 0.104 | -0.395 | 0.179 | -0.115 | 0.021 | -0.107 |

| **(b)** | | | | | | |
| --- | --- | --- | --- | --- | --- | --- |
| **locus** | **RumaA-2015** | **RumaA-2006** | **RumaA-2005** | **RumaB-2005** | **RumaC-2005** | **RumaB-2003** |
| GpC5b | 0.151 | -0.383 | -0.286 | 0.561 | -0.113 | -0.025 |
| GmmK22 | 0.067 | -0.185 | -0.149 | 0.386 | -0.047 | 0.016 |
| GmmC17 | 0.360 | 0.453 | n/a | n/a | n/a | -0.055 |
| GmmL11 | -0.018 | 0.386 | n/a | 0.654 | 0.640 | 0.136 |
| GmmA06 | -0.013 | 0.186 | 0.043 | 0.217 | 0.363 | -0.042 |
| GpB20b | 0.091 | -0.086 | 0.020 | -0.077 | 0.119 | -0.018 |
| GpC10b | n/a | -0.007 | -0.241 | -0.302 | -0.108 | -0.139 |
| GpA19a | 0.327 | 0.169 | 0.654 | 0.327 | 0.660 | 0.148 |
| GpCAG133 | n/a | -0.018 | n/a | n/a | n/a | n/a |
| GpC26b | 0.120 | -0.252 | 0.174 | 0.027 | 0.107 | 0.027 |

**Table S6.** Pairwise locus *P*-values after testing for linkage disequilibrium estimated in Genepop v4.1 [43].

|  | **Nguruman Escarpment** | **Ruma National Park** |
| --- | --- | --- |
| **Locus pair** | **p-value** | **p-value** |
| GmmA06 & GpC26b | 0.524 | 0.012 |
| GpC5b & GpC26b | 0.289 | 0.125 |
| GmmL11 & GpC26b | 0.910 | 0.194 |
| GmmC17 & GmmK22 | 0.063 | 0.213 |
| GpC5b & GpC10b | 0.239 | 0.233 |
| GmmL11 & GmmA06 | 0.892 | 0.289 |
| GpCAG133 & GpA19a | 0.209 | 0.292 |
| GpC5b & GpB20b | 0.966 | 0.333 |
| GmmL11 & GpB20b | 0.573 | 0.398 |
| GpCAG133 & GmmA06 | 0.472 | 0.454 |
| GpC10b & GpB20b | 0.062 | 0.522 |
| GmmC17 & GpC10b | 0.582 | 0.525 |
| GmmL11 & GpA19a | 0.028 | 0.528 |
| GpCAG133 & GpC5b | 0.766 | 0.551 |
| GmmK22 & GpCAG133 | 0.153 | 0.552 |
| GmmK22 & GpC5b | 0.188 | 0.565 |
| GmmK22 & GpA19a | 0.668 | 0.573 |
| GmmC17 & GmmL11 | 0.841 | 0.648 |
| GpCAG133 & GpC10b | 0.255 | 0.702 |
| GmmA06 & GpA19a | 0.088 | 0.725 |
| GmmL11 & GpC5b | 0.042 | 0.731 |
| GpC10b & GpC26b | 0.279 | 0.744 |
| GmmA06 & GpC5b | 0.732 | 0.776 |
| GpA19a & GpC26b | 0.771 | 0.786 |
| GmmC17 & GpA19a | 0.033 | 0.813 |
| GpA19a & GpC10b | 0.971 | 0.814 |
| GmmK22 & GpB20b | 0.474 | 0.842 |
| GmmA06 & GpC10b | 0.933 | 0.873 |
| GpCAG133 & GpB20b | 0.172 | 0.896 |
| GmmC17 & GmmA06 | 0.314 | 0.897 |
| GmmK22 & GmmA06 | 0.161 | 0.914 |
| GmmC17 & GpC5b | 0.038 | 0.916 |
| GmmC17 & GpC26b | 0.234 | 0.939 |
| GmmL11 & GpC10b | 0.351 | 0.944 |
| GpB20b & GpC26b | 0.493 | 0.946 |
| GpC5b & GpA19a | 0.745 | 0.952 |
| GmmK22 & GpC10b | 0.768 | 0.957 |
| GmmK22 & GpC26b | 0.141 | 0.959 |
| GmmC17 & GpB20b | 0.931 | 0.980 |
| GmmA06 & GpB20b | 0.165 | 0.985 |
| GpA19a & GpB20b | 0.007 | 0.998 |
| GmmK22 & GmmL11 | 0.714 | 0.998 |
| GmmC17 & GpCAG133 | 0.696 | 1.000 |
| GpCAG133 & GmmL11 | 0.667 | 1.000 |
| GpCAG133 & GpC26b | 0.190 | 1.000 |

**Table S7.** Per-sample **(a)** estimates of effective population size (N_e_) based on the Jorde/Ryman temporal method showing the interval used in generations (I), the N_e_ estimate, the 95% CI, and (**b)** bottleneck results showing the *P*-values (*P)* under the two-phase model (TPM), the infinite allele model (IAM) and using the mode-shift test.

| **(a)** | | | |  |  |
| --- | --- | --- | --- | --- | --- |
| **Region** | **Locality** | **Collections Used** | **I** | **N_e_** | **CI** |
| Nguruman | LEN | 2003 to 2015 | 55.8 | 341.7 | 197.6-524.9 |
| Nguruman | LEN | 2009 to 2015 | 26.3 | 148.8 | 89.7-222.7 |
| Nguruman | LEN | 2003 to 2009 | 29.6 | 618.9 | 357.8-950.6 |
| Nguruman | MOK | 2003 to 2015 | 55.8 | 279.6 | 176.4-406.2 |
| Nguruman | PAK | 2003 to 2015 | 55.8 | 314.7 | 174.2-496.2 |
| Ruma | A | 2005 to 2015 | 50.4 | 2568.9 | 1437.8-4023.8 |
| Ruma | A | 2005 to 2006 | 5.0 | 41.7 | 24.5-63.3 |
| Ruma | A | 2006 to 2015 | 45.4 | 401.9 | 242.2-601.5 |
| Ruma | B | 2003 to 2005 | 12.5 | 1753.0 | 992.4-2726.7 |
| **(b)** | | |  |  |  |
| **Region** | **Locality** | **Collections used** | **TPM *P*** | **IAM *P*** | **Mode-Shift** |
| Nguruman | LEN | 2015 | **0.003** | **0.000** | L |
| Nguruman | LEN | 2009 | 0.539 | 0.188 | L |
| Nguruman | LEN | 2003 | 0.116 | **0.012** | L |
| Nguruman | MOK | 2015 | 0.577 | 0.053 | L |
| Nguruman | MOK | 2003 | 0.423 | 0.080 | L |
| Nguruman | PAK | 2015 | 0.116 | **0.012** | L |
| Nguruman | PAK | 2003 | **0.042** | **0.042** | L |
| Ruma | A | 2015 | 0.787 | 0.545 | L |
| Ruma | A | 2006 | 0.947 | 0.722 | L |
| Ruma | A | 2005 | 0.633 | 0.410 | L |
| Ruma | B | 2005 | 0.633 | 0.326 | L |
| Ruma | C | 2005 | 0.680 | 0.320 | L |
| Ruma | B | 2003 | 0.787 | 0.326 | L |

**
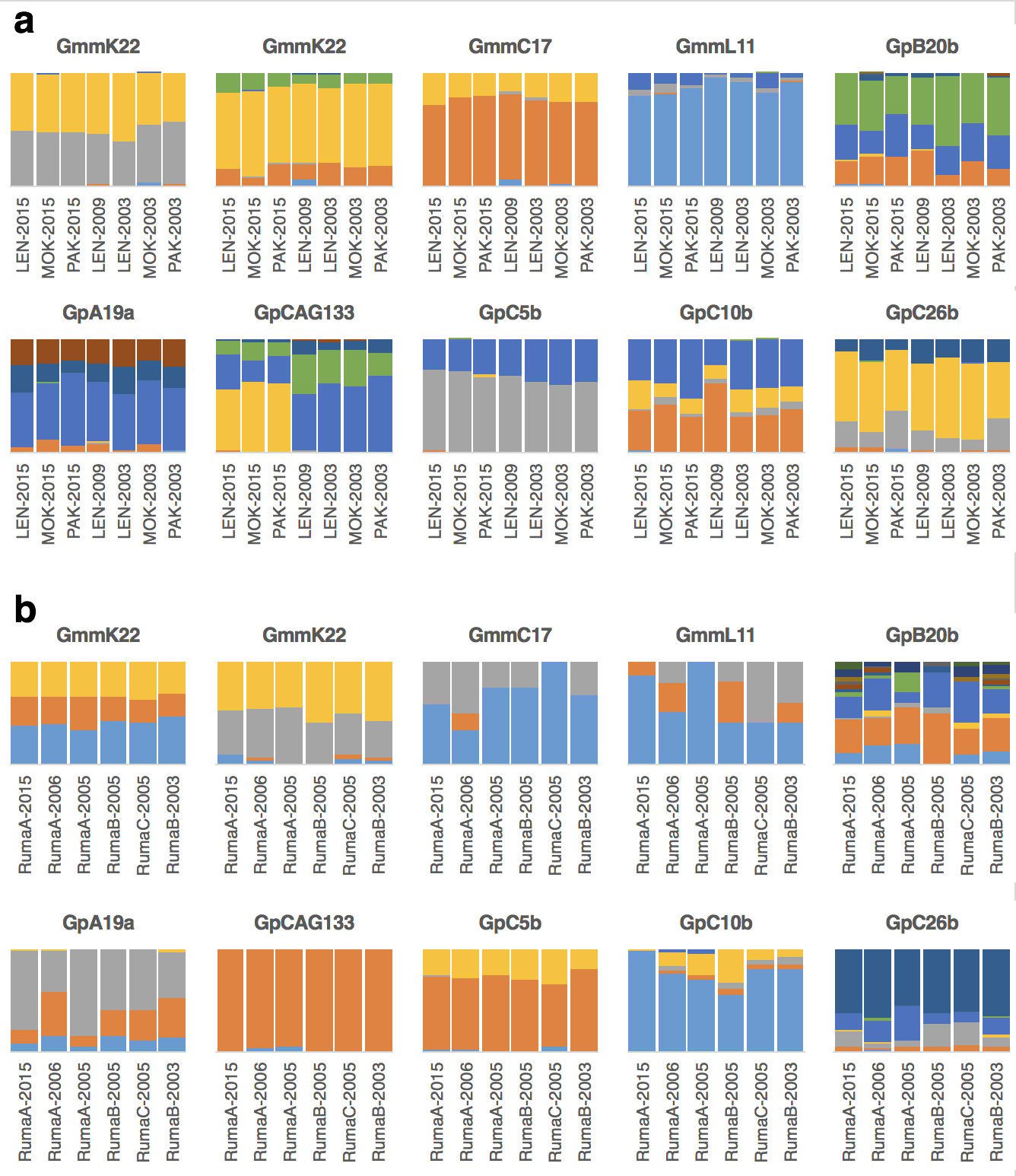
**

**Figure S1**. Allele frequency of each of the 10 microsatellites from **(a)** Nguruman and **(b)** Ruma.

**
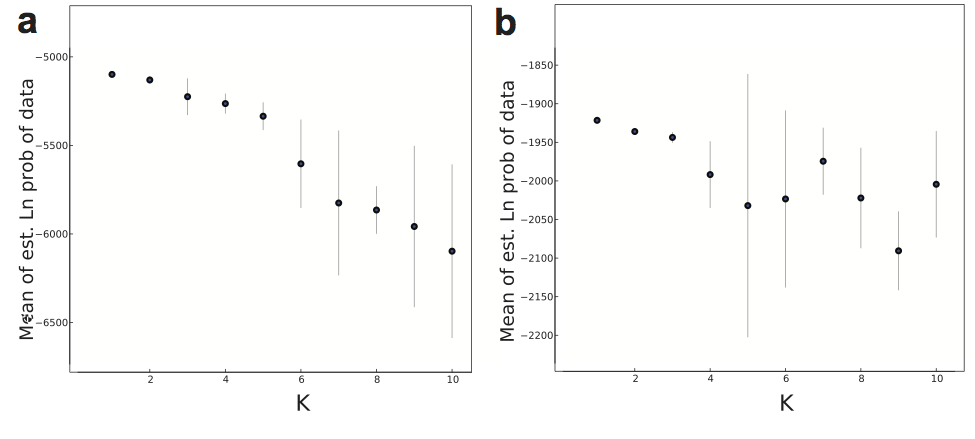
Figure S2**. STRUCTURE [54] estimated Ln likelihood of the data for K values 1–10 for **(a)** Nguruman and **(b)** Ruma, and plots of structure assignment for K values 2–10 for **(c)** Nguruman and (**d)** Ruma.


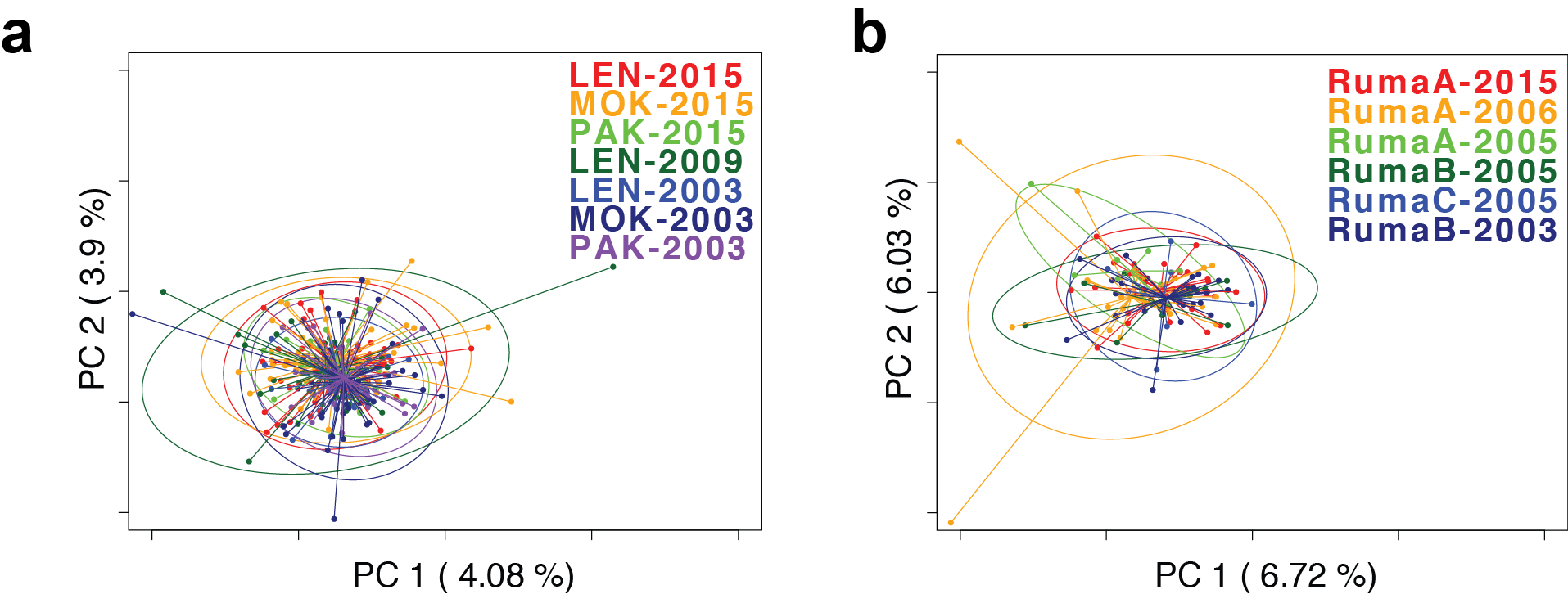


**Figure S3.** Principal Components Analysis (PCA) of microsatellite data from **(a)** Nguruman and **(b)** Ruma.

**
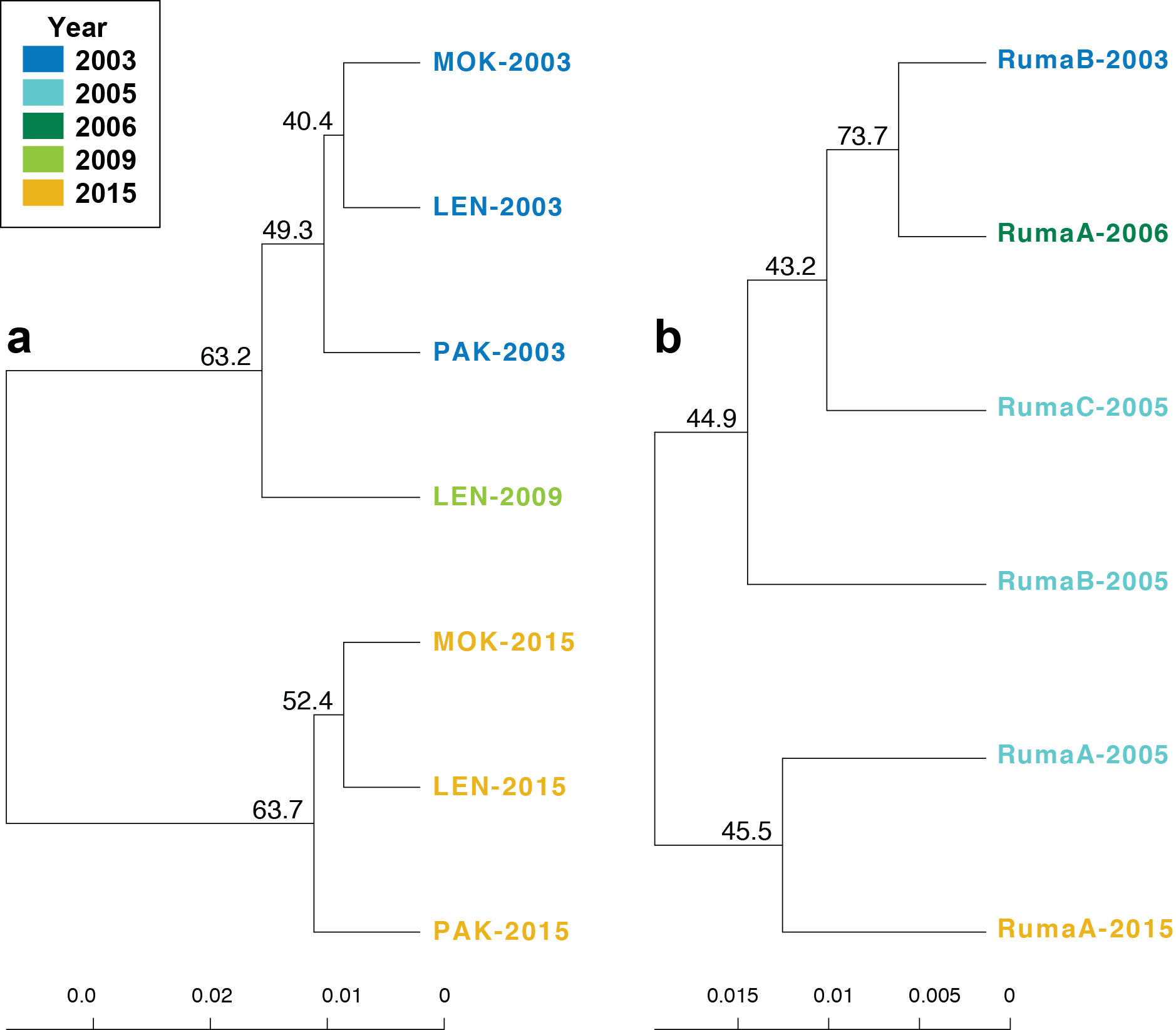
**

**Figure S4.** Neighbor-joining tree [57] using Nei’s genotype frequency based distances **(a)** Nguruman and **(b)** Ruma.
